# Surface‐Anchored Nanogel Coating Endows Stem Cells with Stress Resistance and Reparative Potency via Turning Down the Cytokine‐Receptor Binding Pathways

**DOI:** 10.1002/advs.202003348

**Published:** 2021-01-06

**Authors:** Ling Zhang, Guowu Liu, Kaiqi Lv, Jinxia Xin, Yingchao Wang, Jing Zhao, Wangxing Hu, Changchen Xiao, Keyang Zhu, Lianlian Zhu, Jinliang Nan, Ye Feng, Huaying Zhu, Wei Chen, Wei Zhu, Jianyi Zhang, Jian'an Wang, Ben Wang, Xinyang Hu

**Affiliations:** ^1^ Department of Cardiology, The Second Affiliated Hospital Zhejiang University School of Medicine Hangzhou 310009 China; ^2^ Cardiovascular Key Laboratory of Zhejiang Province Hangzhou 310009 China; ^3^ College of Life Science Zhejiang Chinese Medical University Hangzhou 310053 China; ^4^ Cancer Institute (Key Laboratory of Cancer Prevention and Intervention National Ministry of Education), The Second Affiliated Hospital Zhejiang University School of Medicine Hangzhou 310009 China; ^5^ Institute of Translational Medicine Zhejiang University Hangzhou 310029 China; ^6^ Zhejiang University School of Medicine Hangzhou 310058 China; ^7^ Department of Biomedical Engineering University of Alabama at Birmingham Birmingham AL 35294 USA

**Keywords:** cell surface engineering, cell therapy, engraftment, heart repair, stem cells

## Abstract

Stem cell‐based therapy has great potential in regenerative medicine. However, the survival and engraftment rates of transplanted stem cells in disease regions are poor and limit the effectiveness of cell therapy due to the fragility of stem cells. Here, an approach involving a single‐cell coating of surface‐anchored nanogel to regulate stem cell fate with anti‐apoptosis capacity in the hypoxic and ischemic environment of infarcted hearts is developed for the first time. A polysialic acid‐based system is used to anchor microbial transglutaminase to the external surface of the cell membrane, where it catalyzes the crosslinking of gelatin. The single‐cell coating with surface‐anchored nanogel endows mesenchymal stem cells (MSCs) with stress resistance by blocking the activity of apoptotic cytokines including the binding of tumor necrosis factor α (TNFα) to tumor necrosis factor receptor, which in turn maintains mitochondrial integrity, function and protects MSCs from TNF*α*‐induces apoptosis. The administration of surface engineered MSCs to hearts results in significant improvements in engraftment, cardiac function, infarct size, and vascularity compared with using uncoated MSCs in treating myocardial infarction. The surface‐anchored, biocompatible cell surface engineering with nanogel armor provides a new way to produce robust therapeutic stem cells and may explore immense potentials in cell‐based therapy.

## Graphical abstract

The cell surface masking of a nanogel endows individual cells with enhanced stress resistance and serves as a protective barrier to protect stem cells from apoptosis by reducing the binding between cytokines and their receptors, and improving the reparative potency of transplanted cells through paracrine effects in infarcted hearts, which will aid the development of approaches for cell‐based therapy.

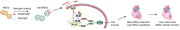

## 1 Introduction

Stem cell‐based therapy is a promising approach for the treatment of cardiovascular disease, diabetes, and other disorders.^[^ ^1^ ^]^ Bone marrow mesenchymal stem cells (MSCs) are among the most common types of cells used for cell therapy because they are self‐renewing and multipotent and can reduce both the immune response and inflammation.^[^ ^2^ ^]^ The results from clinical trials suggest that MSC therapy is safe and feasible^[^ ^3^ ^]^ and may reduce infarct sizes and improve heart function when administered to patients with myocardial infarction (MI).^[^ ^4^ ^]^ However, most cells die within 1 week of transplantation, which limits the effectiveness of the treatment.^[^ ^5^ ^]^ Thus, methods to improve the survival (i.e., the engraftment rate) of transplanted cells are needed to increase the effectiveness of cell therapy. Hypoxia^[^ ^6^ ^]^ or biologically active molecules^[^ ^7^ ^]^ preconditioning is considered as one of effective approach to improve the cell survival, however, hypoxic preconditioning can be tolerated by only a limited range of cell types, and the effect of preconditioning is transient. Although a variety of genetic modifications appear to improve the engraftment and therapeutic potency of MSCs,^[^ ^8^ ^]^ the clinical use of genetically modified cells is limited by concerns about tumorigenicity and immunogenicity.^[^ ^9^ ^]^

Yeast cells can withstand exceptionally harsh environments because they are protected by a shell of organic and inorganic material,^[^ ^10^ ^]^ this observation suggests that the survival potency of transplanted MSCs may be improved if the surfaces of the cells are coated with a shell of biocompatible material. Previous studies have shown that encapsulation of single cells in a thin hydrogel layer could regulate cell differentiation and delay cell clearance.^[^ ^11^ ^]^ However, whether cell surface coating can improve the survival potency of stem cells remains unknown. Existing approaches to encapsulate cells were achieved by deposition of nanolayers on cell surface by electrostatic interactions,^[^ ^12^ ^]^ receptor‐ligand binding,^[^ ^13^ ^]^ polyphenol supramolecular assembly,^[^ ^14^ ^]^ which lead to cell cytotoxicity due to the charge accumulations during layer‐by‐layer assembly of polyelectrolytes on the cell membrane.^[^ ^15^ ^]^ Meanwhile, cell proliferation is hindered by microgel encapsulation due to the complete package with an overly thick hydrogel layer.^[^ ^16^ ^]^ Furthermore, how the coating influences cellular functions is unclear; thus far, there has been little work to illustrate the underlying mechanism of hydrogel coating on the cell function, either in vitro or in vivo. Therefore, it is of long‐term significance to establish a cell coating approach to enhance the cell survival and maintain cell proliferation, as well as understand the mechanism of cell surface engineering for regulating cell fate.

Here, we introduce a principle of using surface‐anchored nanogel to produce robust therapeutic cells by using a polysialic acid‐based system to anchor microbial transglutaminase (mTG) to the cell membrane, where it catalyzes the formation of a nanogel coating over the surface of individual MSCs, this biocompatible approach endows therapeutic stem cells with cell vitality, stress resistance, and without altering cellular functions for the first time. Furthermore, we demonstrate a first mechanism understanding about increasing the survival capacity of nanogel coated cells through shutting down pro‐apoptotic pathway. We reveal that the surface nanogel coating is not a niche microenvironment^[^ ^11^ ^]^ or tool for immune‐isolation,^[^ ^17^ ^]^ but serve as a moderator of cellular pathways and protect the MSCs from apoptosis by reducing the binding between cytokines and their receptors, preserving mitochondrial function through the I‐kappa‐B (I*κ*B)/nuclear factor‐ kappa B (NF*κ*B)/optic atrophy (OPA)1 and peroxisome proliferator‐activated receptor‐γ co‐activator (PGC)‐1*α*/mitofusin (MFN)2 pathways without substantially altering the multipotency or cytokine production of the cells, and further improving the reparative potency of transplanted cells through paracrine effects in infarcted hearts, which will aid the development of novel approaches for cell‐based therapy.

## 2 Results

### 2.1 Individual MSCs Can be Coated with Hydrogel by Anchoring mTG to the Cell Surface with a Polysialic‐Acid Construct

**Figure 1:**
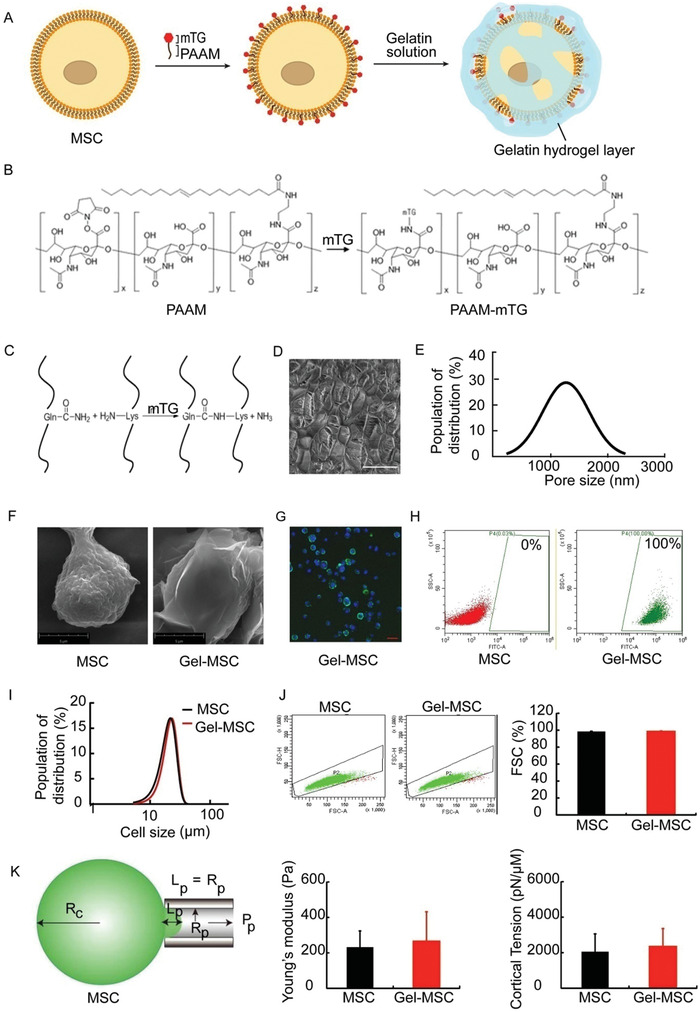
The PAAM‐mTG method efficiently generates a nanogel coating over the surface of individual MSCs. A) The PAAM domain inserts into the cell membrane, leaving the mTG moiety exposed on the surface, where it catalyzes the polymerization of nanogel molecules. B) Polysialic acid was linked to an olyel chain (left) and then reacted with mTG to generate a PAAM‐mTG molecule (right). C) The *γ*‐carboxamide group of glutamine functions as the acyl donor, and the *ε*‐amino group of lysine usually functions as the acyl acceptor. The polymerized nanogel D) formed in bulk or F) coating on the individual MSCs. Bar = 5 µm. E) The pore size distribution of cross‐linked gelatin. G) Gel‐MSCs were viewed via fluorescent microscope (green: FITC‐labeled nanogel, blue: Hoechst‐stained nuclei; bar = 20 µm). H) The proportion of cells that were coated by the nanogel. I)The size of Gel‐MSCs, *n* = 6. J) Cell aggregation was detected and quantified by FACS, *n* = 3. K) The mechanical properties of uncoated MSCs and Gel‐MSCs was evaluated via micropipette aspiration (left panel) and compared by calculating the Young's modulus (center panel) and cortical tension (right panel).

**Figure 2:**
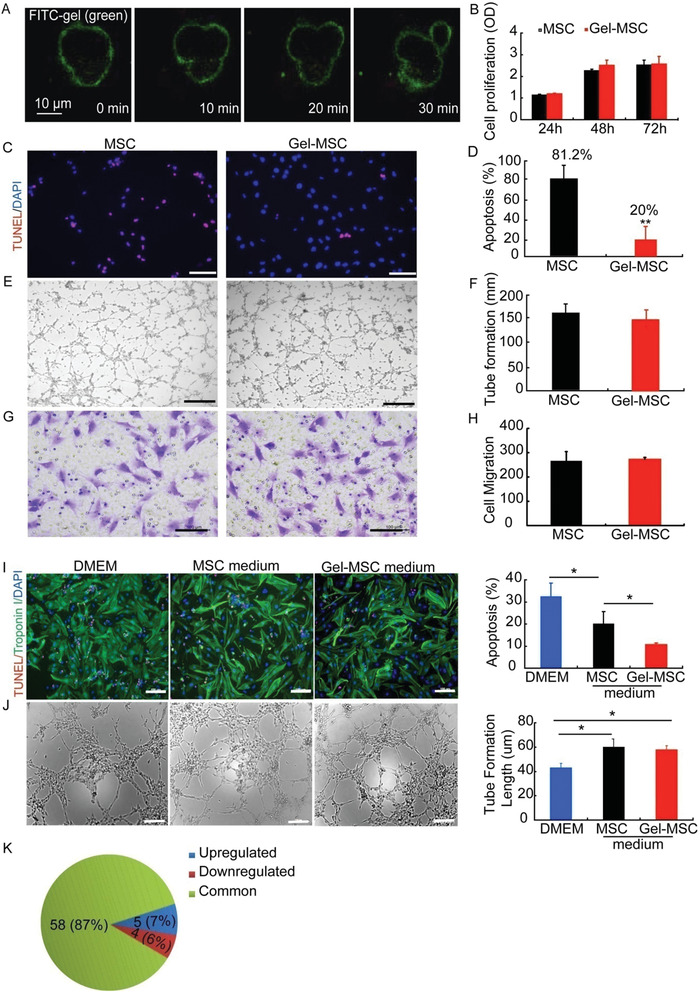
Cell surface nanogel coating protects cultured MSCs from hypoxia‐induced apoptosis. A)The distribution of gelatin coated MSCs during their division (bar = 10 µm). B) Cell proliferation of MSCs and Gel‐MSCs, *n* = 3. C,D) Cell viability and apoptosis assay of MSCs and Gel‐MSCs cultured in serum‐ and sugar‐free medium under hypoxic conditions (bar = 100 µm), *n* = 3. E,F) Tube formation of MSCs and Gel‐MSCs (bar = 500 µm), *n* = 3. G,H) Cell migration of MSC and Gel‐MSCs (bar = 100 µm), *n* = 3. I) Apoptosis of neonatal rat cardiomyocytes cultured with standard media (DMEM) or with media collected from MSCs or Gel‐MSCs under hypoxic conditions evaluated via terminal deoxynucleotidyltransferasedUTP nick end labeling (TUNEL) staining (bar = 100 µm), *n* = 4. J) Tube formation of HUVECs with DMEM or with media collected from MSCs or Gel‐MSCs (bar = 100 µm), *n* = 3. K) The levels of expression for 67 cytokines in MSCs and Gel‐MSCs were compared via microchip analysis. DAPI, 4',6‐diamidino‐2‐phenylindole.

An oleyl chain was grafted onto polysialic acid to generate a polysialic acid anchor for cell membranes (PAAM); then, the PAAM was linked to microbial transglutaminase mTG in the presence of *N*‐hydroxysuccinimide to form the PAAM‐mTG molecule (**Figure** **1**A and FigureS1A,C, Supporting Information). The PAAM was inserted into the lipid bilayer, leaving the mTG moiety exposed on the exterior surface (Figure 1B), where it catalyzed the crosslinking of adjacent gelatin molecules (Figure 1C; FigureS2A, Supporting Information) to form the nanogel coating (Figure 1D). The average pore size of nanogel coating is about 1.2 µm characterized by cryo‐scanning electron microscope (cryo‐SEM) (Figure 1E). Both SEM and transmission electron microscopy (TEM) showed that the cell surface morphology of nanogel coated MSCs was smoother than that in bare MSCs (Figure 1F and Figure S2B, Supporting Information). The nanogel on the cell surface was observed via super‐resolution structured illumination microscopy and the thickness of the nanogel coating on the MSCs was appeared to be 332.0 ± 85.8 nm (**Figure** **2**C, Supporting Information). Confocal laser microscopy (Figure 1G) and flow cytometry assessments (Figure 1H) of fluorescein isothiocyanate isomer (FITC) fluorescence, which was conjugated to the gelatin, revealed that up to 100% of MSCs were coated with the nanogel. The average cell sizes before and after nanogel coating were 21.73 and 23.93 µm, respectively (Figure 1I). The relative size of cells determined by flow cytometry showed that there were no differences between MSCs and Gel‐MSCs as represented by forward scatter (FSC) (Figure 1J), indicating that there was no significant cell aggregation during gelation. Furthermore, the measures of Young's modulus and cortical tension (Figure 1K) in nanogel‐coated MSCs and uncoated MSCs were equivalent, suggesting that the cell surface nanogel coating did not alter the elasticity or other mechanical properties of the cells.

### 2.2 Cell Surface Nanogel Coating Reduces MSCs Apoptosis without Substantially Altering Cellular Functional Activity

**Figure 3:**
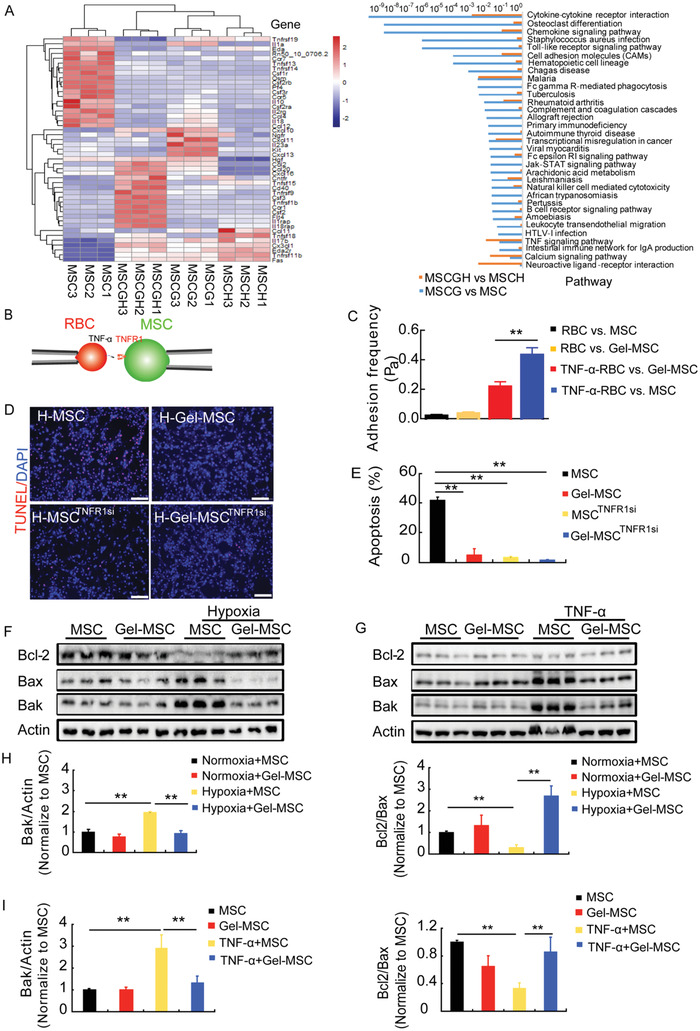
Anti‐apoptotic effect of the cell surface nanogel coating may be mediated by declines in TNF*α* signaling. A) The gene expression profile (i.e., transcriptome) of hypoxia or normoxia‐cultured MSCs and Gel‐MSCs were compared via hierarchical clustering analysis to identify signaling pathways that were significantly changed in Gel‐MSCs. B) Red blood cell (RBC) was coupled to biotinylated TNF*α* and held in contact with MSCs or Gel‐MSCs for 2 s; then C) the force required to separate the cells was measured. D,E) Apoptosis assay ofMSCs, Gel‐MSCs, and MSCs, Gel‐MSCs that had been transfected with TNFR1 siRNA (MSC^TNFRsi^ and Gel‐MSC^TNFRsi^, respectively) when cultured under hypoxic conditions (bar = 100 µm), *n* = 3. F,G) Bcl‐2, Bax, and Bak protein levels in MSCs and Gel‐MSCs under standard and hypoxic conditions or in the absence and presence of TNF*α*. H,I) Bcl‐2/Bax and Bak was quantified in MSCs and Gel‐MSCs that had been cultured under standard and hypoxic conditions or in the absence and presence of TNF*α*, *n* = 3.

The effect of the cell surface nanogel coating on MSC functional activity was evaluated in vitro through a series of assessments with nanogel‐coated MSCs (Gel‐MSCs) and uncoated MSCs. The cells could still divide (Figure 2A) and proliferate (Figure 2B) after being coated with nanogel. The proportion of apoptotic cells was lower in Gel‐MSCs (20%) than in uncoated MSCs (81.2%) that had been cultured under hypoxic conditions in serum‐free medium for 24 h (Figure 2C,D), the protective effect of nanogel was weakened with the proliferation of cells, but it still played a certain role in anti‐apoptosis (FigureS3A,B, Supporting Information). Measures of tube formation (Figure 2E,F), migration (Figure 2G,H), and differentiation (FigureS3C, Supporting Information) in the two MSC populations were equivalent. Furthermore, we compared the cytokine production profiles of Gel‐MSCs and uncoated MSCs, as well as the effect of conditioned medium from the two MSC populations on cardiomyocyte apoptosis and tube formation in human umbilical‐vein endothelial cells (HUVECs). Conditioned medium from both MSC populations protected cardiomyocytes from hypoxia‐induced apoptosis (Figure 2I) and promoted tube formation in HUVECs similarly (Figure 2J). Microchip analysis indicated that only 13% of evaluated cytokines (9 of 67) were expressed at higher (intercellular cell adhesion molecule‐1 (ICAM‐1), chemokine (C‐X‐C motif) ligand 6 (LIX), cluster of differentiation 80 (B7‐1), hepatocyte growth factor (HGF), neuropilin‐2) or lower (cluster of differentiation 86 (B7‐2), granulocyte‐macrophage colony‐stimulating factor (GM‐CSF), interleukin‐4 (IL‐4), platelet‐derived growth factor AA (PDGF‐AA)) levels in Gel‐MSCs than in MSCs (Figure 2K). The up‐ or downregulated cytokines were then confirmed by enzyme linked immunosorbent assay (ELISA), the concentrations of IL‐4, HGF, and PDGFAA were consistent with microchip analysis, however the other cytokines remained unchanged (FigureS3D, Supporting Information). Furthermore, the nanogel coating did not affect the exosome (average particle size: 138.3 nm) uptake by MSCs (FigureS3E,F, Supporting Information). These findings suggest that the nanogel coating did not change the cytokine secretion and nutrient absorption activities of MSCs.

### 2.3 Protective Effect of the Cell Surface Nanogel Coating on MSC Survival is Mediated by Decreases in Cytokine and Cytokine Receptor Binding

To explore the mechanism for the enhanced survival of Gel‐MSC, we investigated the transcriptome of MSC‐Gel and MSCs under hypoxia and normoxia condition. Pathway analysis identified 33 molecular pathways that appeared to be substantially altered in Gel‐MSCs compared with uncoated MSCs (**Figure** **3**A) in normoxia and hypoxia, and the expression of genes involved in cytokine–cytokine receptor interaction pathways showed the most significant differences in Gel‐MSC compared to MSC under both hypoxia and normoxia (Figure 3A). Because the severe inflammation in the heart after MI is an important factor affecting transplanted cell survival, we therefore hypothesized that the nanogel coating may improve transplanted cell survival by reducing cytokine–cytokine receptor interaction. Tumor necrosis factor (TNF) signaling, which is known to play an important role in cell survival,^[^ ^18^ ^]^ was also significantly changed in Gel‐MSCs. Thus, we determined whether the cytoprotective effects of the nanogel coating were attributed, at least in part, to a decrease in the interaction between TNF*α* and the TNF receptor (TNFR), which in turn attenuated TNF*α* signaling. Measurements of the force of binding between TNF*α* and the TNFR were significantly lower in Gel‐MSCs than in uncoated MSCs (Figure 3B,C). The proportion of TNFR1 was sevenfold higher than that of TNFR2 by quantitative real‐time polymerase chain reaction (qPCR) measurement (Figure S4A, Supporting Information), indicating that the dominating effect of TNF*α* occurred through TNFR1. By knocking‐down the TNFR1 with siRNA (Figure S4B, Supporting Information), we showed that the decrease in apoptosis associated with TNFR1 siRNA recapitulated the protective effects of the nanogel coating when the cells were exposed to hypoxia (Figure 3D and 3E). Hypoxia (Figure 3F,H) and/or treatment with TNF*α* (Figure 3G,I) also substantially altered the expression of a number of apoptotic proteins in MSCs: anti‐apoptotic protein (B‐cell lymphoma‐2 (Bcl2)) levels decreased while the levels of pro‐apoptotic proteins (B‐cell lymphoma‐2 associated X (Bax) and B‐cell lymphoma‐2 homologous antagonist/killer (Bak)) levels increased under both conditions, but this did not occur (or to a lesser extent) in Gel‐MSCs. Collectively, these observations suggest that the cell surface nanogel coating protects MSCs from hypoxia‐induced apoptosis partially by disrupting the binding of TNF*α* to the TNFR.

### 2.4 Decreased Binding Between TNF*α* and its Receptor Caused by Cell Surface Nanogel Coating Maintains Mitochondrial Integrity and Improves Mitochondrial Function

**Figure 4:**
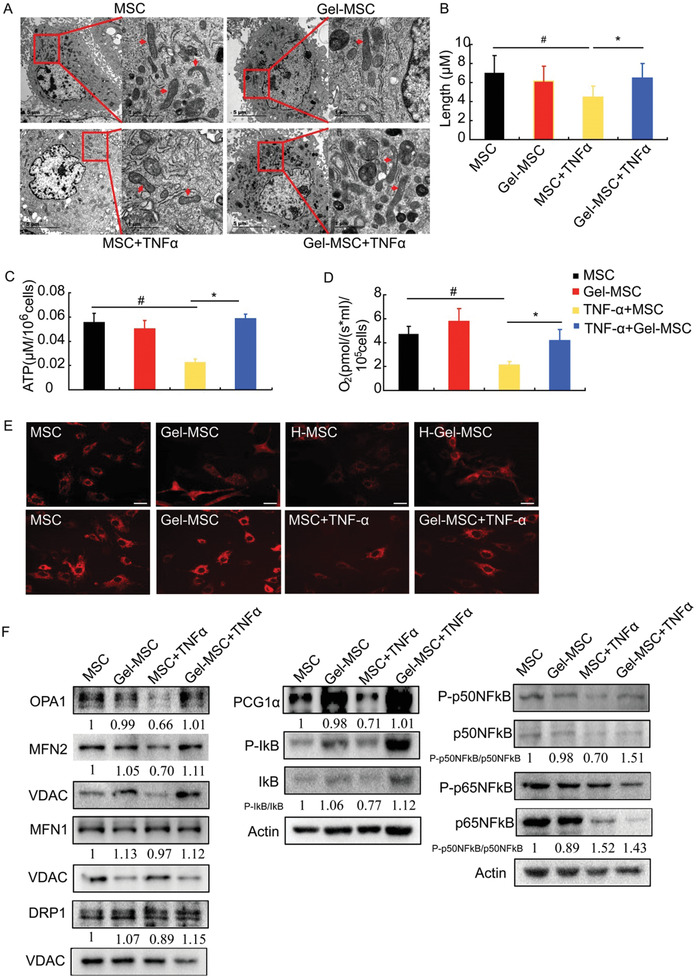
The cell surface nanogel coating maintains mitochondrial integrity and improves mitochondrial function of MSCs after TNF*α* stimulation. A) Mitochondrial ultrastructures were detected by TEM to show the mitochondrial length (left: bar = 5 µm, right: bar = 1 µm). B) The mitochondrial length was measured at least five cells for each group. C) Intracellular ATP levels were evaluated in MSCs and Gel‐MSCs that had been cultured under standard and hypoxic conditions (left) or in the absence and presence of TNF*α* (right) (*n* = 3). D) Maximum oxygen consumption rates were evaluated in MSCs and Gel‐MSCs that had been cultured under standard and hypoxic conditions (left) or in the absence and presence of TNF*α* (right) (*n* = 3). E) Mitochondrial membrane potentials were evaluated via TMRM staining in MSCs and Gel‐MSCs that had been cultured under standard and hypoxic (H‐MSC and H‐Gel‐MSC) conditions or in the absence and presence of TNF*α*. F) Critical protein levels were evaluated via Western blot in MSCs and Gel‐MSCs in the absence and presence of TNF*α*; voltage dependent anion channel (VDAC, mitochondria) and actin (cytosol) levels were evaluated to confirm the equal loading, the values of OPA1, MFN2, MFN1, DRP1 are the ratio of VDAC; the value for PGC1*α* is the ratio with actin, and the values for P‐IκB, P‐p50NFκB, P‐p65NFκB are the ratios with IκB, p50NFκB, and p65NFκB respectively. *n* = 3. ^#^ *P* < 0.05 versus MSC; * *P* < 0.05 versus TNF*α*+MSC.

To determine whether nanogel coating affected mitochondrial integrity after exposure to TNF*α*, transmission electron microscopy was used to analyze the mitochondrial length. The mitochondrial length was significantly shorter in MSCs after exposure to TNF*α* than under normal conditions, while the mitochondrial length was longer in Gel‐MSCs than in MSCs after exposure to TNF*α* (**Figure** **4**A,B). Accordingly, the integrity changes induced by TNF*α* treatment resulted in significant decreases in adenosine triphosphate (ATP) levels (Figure 4C) and the rate of oxygen consumption (Figure 4D); however, these changes were much lower in Gel‐MSCs than in uncoated MSCs, and the same pattern of changes was observed for mitochondrial membrane potentials when comparing Gel‐MSCs versus uncoated MSCs after exposure to TNF*α* (Figure 4E). These findings indicate that nanogel coating maintained the mitochondrial integrity and hence improved mitochondrial function.

To determine the underlying mechanism through which nanogel coating maintained the mitochondrial integrity, we analyzed the genes that regulate mitochondrial fusion and fission processes, including OPA1, MFN1, MFN2, and dynamin‐related protein 1 (DRP1). The results showed that there were no changes in the protein expression of DRP1 and MFN1 between the MSC and Gel‐MSC groups under normal conditions or even after exposure to TNF*α*. However, the expression of OPA1 and MFN2 significantly decreased in MSCs after TNF*α* treatment but increased in the Gel‐MSC group compared with that in the MSC group after exposure to TNF*α* (Figure 4F). Furthermore, PGC‐1*α* and phosphorylated I*κ*B/p50NF*κ*B, the upstream molecules of MFN2 andOPA1, respectively, significantly decreased after TNF*α* induction in the MSC group; however, expression increased in the Gel‐MSC group compared with that in the MSC group after exposure to TNF*α* (Figure 4F). These results suggest that PGC‐1*α*/MFN2 and I*κ*B/NF*κ*B/OPA1might be involved in the nanogel coating‐mediated improvement in cell survival.

### 2.5 OPA1 and MFN2 are Required for the Cell Surface Nanogel Coating‐Mediated Mitochondrial Function Improvement

**Figure 5:**
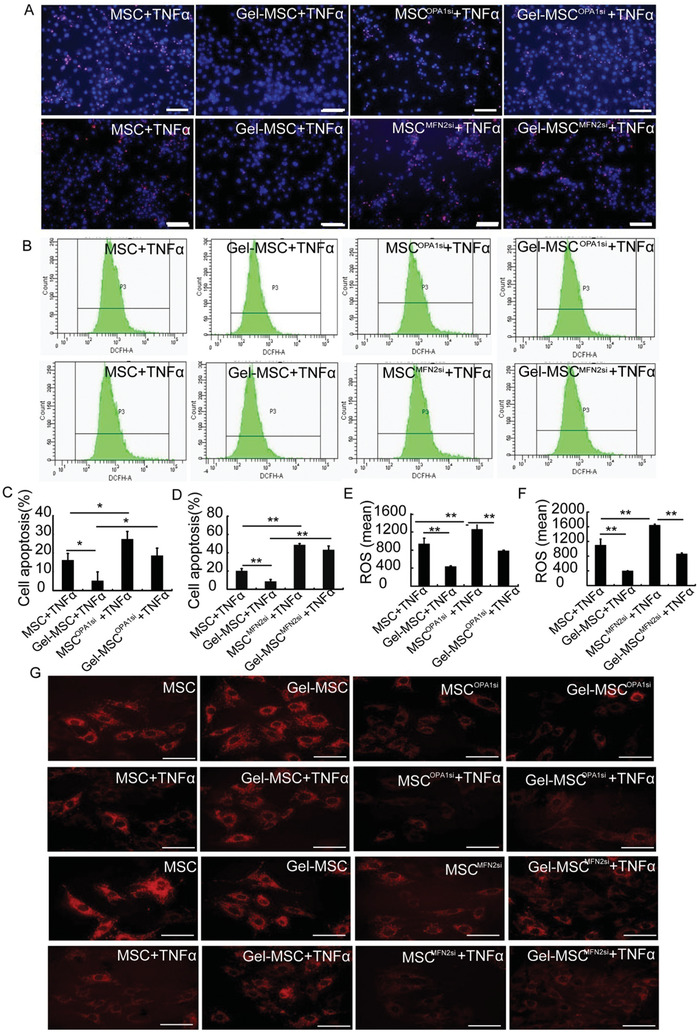
OPA1 and MFN2 were required for protective effects of the cell surface nanogel coating in response to TNF*α* stimulation. A) MSCs, Gel‐MSCs, and MSCs, Gel‐MSCs that had been transfected with OPA1 siRNA (MSC^OPA1si^ and Gel‐MSC^OPA1si^, respectively) and MFN2 siRNA (MSC^MFN2si^ and Gel‐MSC^MFN2si^, respectively) were stimulated with TNF*α*; then, apoptotic cells were identified via TUNEL (red); nuclei were counterstained with DAPI (blue) (bar = 100 µm) and C,D) quantified as the percentage of the total number of cells (*n* = 3). B) ROS production was detected by FACS and E,F) quantified in MSCs, Gel‐MSCs, MSC^OPA1si^, Gel‐MSC^OPA1si^, MSC^MFN2si^, and Gel‐MSC^MFN2si^ in the presence of TNF*α*. G) Mitochondrial membrane potentials were evaluated via TMRM staining in MSCs, Gel‐MSCs, MSC^OPA1si^, Gel‐MSC^OPA1si^, MSC^MFN2si^, and Gel‐MSC^MFN2si^ in the absence and presence of TNF*α*.

To further clarify whether OPA1 and MFN2 are necessary for the effect of the nanogel coating on maintaining mitochondrial function and cell survival, we analyzed cell survival, mitochondrial membrane potentials, and the level of reactive oxygen species (ROS) formation in MSCs with OPA1 or MFN2 knockdown (Figure S4C, Supporting Information). After exposure to TNF*α*, decrease in apoptosis was observed in Gel‐MSCs compared with non‐coating MSCs; however, when OPA1 and MFN2 were knocked down using OPA1 siRNA and MFN2siRNA, the protective effect of the nanogel coating on MSCs was abolished (**Figure** **5**A,C,D). Furthermore, the decreased ROS (Figure 5B,E,F) and increased mitochondrial membrane potentials (Figure 5G) mediated by nanogel coating were reversed when Gel‐MSCs were transfected with OPA1 or MFN2 siRNA. These findings indicate that OPA1 and MFN2 might be required for the cell surface nanogel coating‐mediated improvements in MSC survival and mitochondrial function after TNF*α* stimulation.

### 2.6 Cell Surface Nanogel Coating Improves the Engraftment and Reparative Potency of Transplanted MSCs in Infarcted Rat Hearts

**Figure 6:**
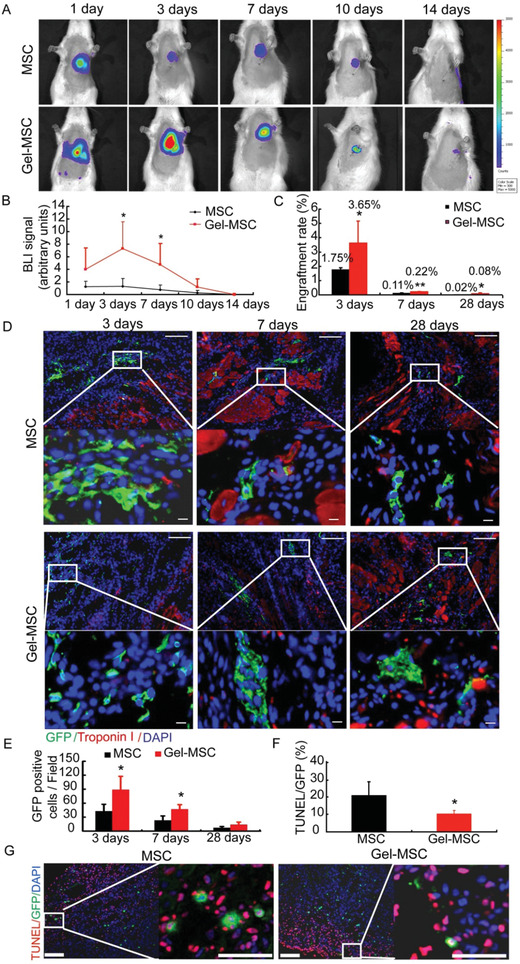
Cell surface nanogel coating improves the engraftment of transplanted MSCs. A) Bioluminescence images of luciferase activity obtained at the indicated time points after MI and treatment, and B) engraftment was quantified as the intensity of the bioluminescence signal. *n* ≥ 4. C) Engraftment was quantified at the indicated time points after MI and treatment via qPCR measurements of GFP expression. *n* ≥ 3. D) Engrafted cells were identified in myocardial tissues via immunofluorescence imaging of GFP expression (middle two rows: bar = 100 µm, top and bottom rows: bar = 10 µm), and E) GFP positive cells were quantified at the indicated time points after MI and treatment, *n* = 3. F) The apoptotic rate of injected MSCs, *n* = 3. G) Apoptosis of injected cells detected by co‐staining of GFP and TUNEL (bar = 100 µm).

**Figure 7:**
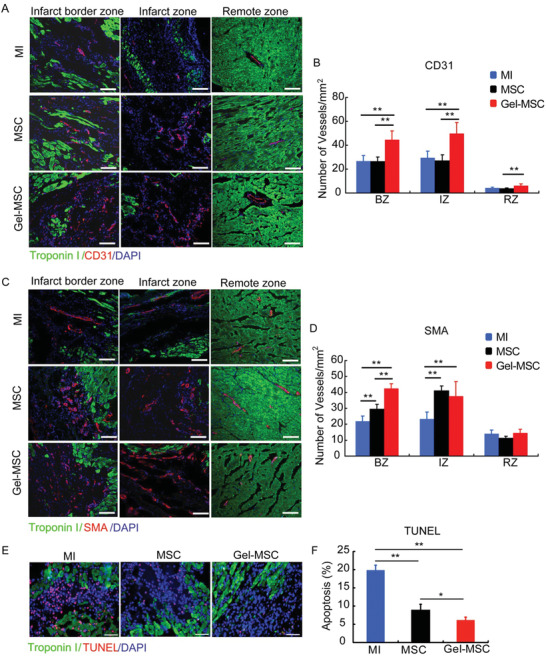
Cell surface nanogel coating improves the reparative potency of MSCs for treatment of MI in rats. A) Sections collected from the border zones of infarction, infarct zones, and remote zones on day 28 were stained for CD31 expression to identify endothelial cells. B) Vascularity was quantified as the number of CD31‐positive structures. C) Smooth muscle actin (SMA) expression to identify smooth‐muscle cells. D) Arteriole density was quantified as the number of SMA‐positive structures. Bar = 100 µm. E) Sections collected from the border zones of infarction on day 3 were stained for TUNEL stained to identify apoptotic cells. F) Apoptosis was quantified as the percentage of TUNEL‐positive cells. Bar = 50 µm. BZ: infarct border zone; IZ: infarct zone; RZ: remote zone.

**Figure 8:**
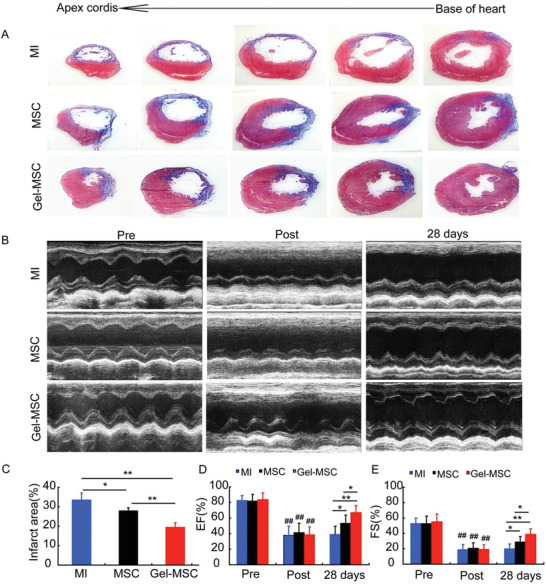
Measures of infarct size and cardiac function in infarcted rat hearts were better after treatment with nanogel‐coated MSCs than with uncoated MSCs. A) The heart was cut into five layers from base of heart to apex cordis 28 days after MI and treatment, and sections of the left ventricles (LV) were stained to identify regions of normal (red) and infarcted (blue) tissue. B) Echocardiographic images were obtained before MI (pre), as well as immediately after (post) and 28 days after the MI induction surgery was complete. C) Histological measurements of infarct size were determined by calculating the proportion of the LV surface that was occupied by the infarct and expressed as a percentage, *n* = 4. D,E) Echocardiographic measurements of LV ejection fraction (EF) and fractional shortening (FS) were determined at the indicated time points, *n* ≥ 8; ^##^ *p*< 0.01 versus pre in the same treatment group.

To determine whether the cytoprotective effect of the nanogel coating observed in vitro may improve the survival and reparative potency of transplanted cells, MI was surgically induced in the hearts of rats, and the animals were treated with Gel‐MSCs (i.e., the Gel‐MSC group) or uncoated MSCs (i.e., the MSC group) 30 min later. The MSCs, which were engineered to express luciferase and green fluorescent protein (GFP), were injected into five sites near the border of the infarct; the surviving cells were quantified at later time points by bioluminescence imaging (BLI) of luciferase activity and qPCR measurements of GFP DNA levels. The BLI signal was significantly greater, by as much as sixfold, in the Gel‐MSC group than in MSC animals on days 3 and 7 after cell administration (**Figure** **6**A,B) but not at later time points, while qPCR assessments indicated that engraftment was significantly greater for Gel‐MSCs than for uncoated MSCs through day 28 (Figure 6C). To detect whether transplanted MSCs remain coated by nanogel or not in vivo, MSCs were coated with sulfo‐cyanine7 (Cy7)‐labeled gelatin and observed by fluorescence microscope, and the results revealed that the nanogel around MSCs can still be detected but decreased from day 3 to 14 after transplantation (Figure S5, Supporting Information). Immunofluorescence assessments of GFP expression on days 3 and 7 also indicated that engrafted MSCs were much higher in the Gel‐MSC group than in MSC group (Figure 6D,E). Co‐staining of GFP and TUNEL revealed that the apoptotic rate of injected MSCs was nearly 21%, while the apoptotic rate of nanogel‐coated MSCs was about 10% at 3 days after injection (Figure 6F,G). Furthermore, measurements of vascular density (**Figure** **7**A,B), arteriole density (Figure 7C,D), infarct size (**Figure** **8**A,C) and cardiac function (left‐ventricular ejection fraction and fractional shortening^[^ ^19^ ^]^) (Figure 8B,D,E) on day 28, as well as apoptosis (Figure 7E,F) on day 3, were significantly better in Gel‐MSC‐treated animals than in MSC‐treated animals and in both cell‐treatment groups than in the MI group. Thus, the cell surface nanogel coating appeared to significantly increase the engraftment and reparative potency of transplanted MSCs in infarcted rat hearts.

## 3 Discussion

The low survival rates of transplanted stem cells are poor and limit the effectiveness of cell therapy. In this study, we provide a biocompatible approach to achieve robust therapeutic stem cells with stress resistance by single‐cell coating of surface‐anchored nanogel for the first time. The nanogel coating improves the cell survival and reparative potency by blocking the cytokine‐cytokine receptor binding, which maintains the mitochondrial integrity and function through the I*κ*B/NF*κ*B/OPA1 and PGC‐1*α*/MFN2 pathways.

The approach developed in this study has several features. First, cell viability and proliferation can be maintained for longer period via the surface‐anchored nanogel masking developed in this study, compared to no cell division in the microgel encapsulation which is complete package of cells with an overly thick hydrogel layer.^[^ ^16^ ^]^ The results confirmed that the single‐cell coating with surface‐anchored nanogel via polysialic acid‐based method could maintain cell viability nicely and further serves as a barrier between the cell and environment to block the activity of apoptotic cytokines and enhance the survival potential of the coated MSCs. This cell surface‐anchored masking approach achieves the cell coating by molecular self‐assembly on the cell membrane without charge accumulation or chemically covalent modification, which is more biocompatible. Second, this polysialic acid‐based method results in the nanoscale coating of hydrogel on the cell membrane to guarantee the cell surface nanogel coating does not alter the elasticity or other mechanical properties of the cells confirmed by the equivalent Young's modulus and cortical tension in nanogel‐coated MSCs and uncoated MSCs. Third, the single‐cell surface coating is found to serve as a moderator of cellular pathways and protect the MSCs from apoptosis by maintaining mitochondrial integrity and function, which is different from providing an environment for cell retention,^[^ ^11^ ^]^ homing,^[^ ^20^ ^]^ and immunoprotecting.^[^ ^17^, ^21^ ^]^ Forth, the nanogel coating did not affect the biological characteristics of MSC, such as, tube formation, migration, and differentiation, most importantly, it did not affect the paracrine effect of MSC, as it has been well known that the therapeutic effect of MSC is mainly through paracrine function, and MSCs are difficult to differentiate into cardiomyocyte in vivo.^[^ ^22^ ^]^Finally, the cell surface‐anchored masking can also be used as a scaffold for chemical and biological modification through conjugation with drug‐loaded cargoes,^[^ ^23^ ^]^ which, in principle, provides functional modules of targeting and survival such as continuous, pseudo‐autocrine stimulation to the therapeutic cells.

Severe inflammation in MI hearts is detrimental to transplanted cell survival. Blocking the inflammation factors in heart tissue from interacting with receptors on MSCs is beneficial to cell survival. The single‐cell nanogel coating can form a barrier between cells and the harsh environment to block the high levels of apoptotic cytokines to bind with their receptors. Our observation that the nanogel coating significantly reduced the force of binding between TNF*α* and TNFR, as well as hypoxia‐induced apoptosis by an amount similar to that observed in the presence of TNFR siRNA, strongly suggests that the higher rate of survival observed in animals treated with Gel‐MSCs than in those treated with uncoated MSCs can be at least partially attributed to the disruption of TNF*α* and TNFR interactions.^[^ ^24^ ^]^ This assertion is also supported by measurements of pro‐apoptotic (bax and bak) and anti‐apoptotic (Bcl‐2) protein levels, which were lower and higher (respectively) in Gel‐MSCs than in uncoated MSCs, and by the higher rates of ATP production and oxygen consumption observed in nanogel‐coated cells.

Mitochondrial integrity and function are important to cell survival. Mitochondria are morphologically dynamic organelles regulated by fusion and fission, and when cells are in the process of apoptosis, the mitochondrial fragments become smaller.^[^ ^25^ ^]^ The results presented here indicated that the single‐cell nanogel coating regulated mitochondrial fusion by increasing OPA1 and MFN2 protein levels in TNF*α*‐stimulated cells. After TNF*α* stimulation, NF*κ*B activation by I*κ*B phosphorylation^[^ ^26^ ^]^ appears to play an essential role in promoting the transcriptional activity of the OPA1 gene, which is considered essential for the maintenance of mitochondrial integrity and protection from stress‐induced cell death.^[^ ^27^ ^]^ MFN2 was reported to be regulated by PGC‐1*α* in TNF*α*‐induced hepatic mitochondrial swelling and cell apoptosis.^[^ ^28^ ^]^ Here, for the first time, we demonstrated that the mechanism of the nanogel coating‐mediated increase in cell survival was associated with reducing the binding between TNF*α* and TNFR, activating I*κ*B/NF*κ*B‐responsive and PGC‐1*α*‐responsive promoter elements, and then transcriptionally upregulating OPA1 and MFN2 to maintain mitochondrial integrity and thereby protect cell from TNF*α*‐induced apoptosis.

## 4 Conclusion

In conclusion, we achieve robust therapeutic stem cells with stress resistance by using a biocompatible, polysialic‐acid based and surface‐anchored nanogel over individual MSCs. The cellular surface nanogel coating is found to serve as a moderator of cellular pathways and protect the MSCs from apoptosis in the hypoxic and ischemic environment of disease region by maintaining mitochondrial integrity and function. Measures of cardiac functional recovery and infarct size were significantly better in rats that were treated with the nanogel‐coated MSCs than in those treated with uncoated MSCs after MI. Additional investigations are warranted to determine whether this same technique can improve the effectiveness of other cell types for use in reparative therapy.

## 5 Experimental Section

Experimental details are provided in Supporting Information. Statistical analysis with one tailed *t*‐tests for comparisons between two groups and one‐way analysis of variance and Tukey correction for comparisons among more than two groups and *p* < 0.05 was considered significant.

## Conflict of Interest

The authors declare no conflict of interest.

## Author Contributions

L.Z., G.L., and K.L. contributed equally to this work. X.H, B.W., and J.W. conceived and designed the experiments. G.L. and J.X. performed the synthesis and characterizations of cell surface nanogel engineering and cell division test. K.L., Y.W., and W.H. carried out the animal experiments and the data analyses. J.Z. performed echocardiography and MSC‐Gel differentiation. C.X. performed TMRM staining and western blot. K.Z. performed qPCR analysis of GFP DNA levels. L.Z., J.N., and L.Z. performed cell oxygen consumption. H.Z. and W.C. carried out mechanical property testing and binding force assay. Y.F. contributed to hierarchical clustering analysis of RNA‐seq. J.Z. (US) and W.Z. contributed to data interpretation and manuscript preparation. L.Z., G.L., X.H, B.W., and J.W. wrote the manuscript and received comments and edits from all the authors.

## Supporting information

Supporting Information
