# Supplementary material for: Surface‐Anchored Nanogel Coating Endows Stem Cells with Stress Resistance and Reparative Potency via Turning Down the Cytokine‐Receptor Binding Pathways
Source: Adv Sci (Weinh). 2021 Jan 6;8(3):2003348. doi: 10.1002/advs.202003348 (PMC7856906; doi:10.1002/advs.202003348)
Supplement: Supplementary file 1 — Supporting Information [file ADVS-8-2003348-s001.pdf]

WILEY-VCH

© 2020 Wiley-VCH GmbH

ADVANCED SCIENCE
Open Access

Supporting Information

for *Adv. Sci.*, DOI: 10.1002/advs.202003348

Surface-Anchored Nanogel Coating Endows Stem Cells with Stress Resistance and Reparative Potency via Turning Down the Cytokine-Receptor Binding Pathways

Ling Zhang, Guowu Liu, Kaiqi Lv, Jinxia Xin, Yingchao Wang, Jing Zhao, Wangxing Hu, Changchen Xiao, Keyang Zhu, Lianlian Zhu, Jinliang Nan, Ye Feng, Huaying Zhu, Wei Chen, Wei Zhu, Jianyi Zhang, Jian'an Wang, Ben Wang, Xinyang Hu**

© 2020 Wiley-VCH GmbH

Supporting Information

Surface-Anchored Nanogel Coating Endows Stem Cells with Stress Resistance and Reparative Potency via Turning Down the Cytokine-Receptor Binding Pathways

Ling Zhang, Guowu Liu, Kaiqi Lv, Jinxia Xin, Yingchao Wang, Jing Zhao, Wangxing Hu, Changchen Xiao, Keyang Zhu, Lianlian Zhu, Jinliang Nan, Ye Feng, Huaying Zhu, Wei Chen, Wei Zhu, Jianyi Zhang, Jian'an Wang, Ben Wang*, Xinyang Hu*

Dr L. Zhang, K. Lv, Y. Wang, J. Zhao, Dr W. Hu, C. Xiao, Dr K. Zhu, L. Zhu, Dr J. Nan, Dr H. Zhu, Prof. W. Chen, Prof. W. Zhu, Prof. J. Wang, Prof. X. Hu
Department of Cardiology, The Second Affiliated Hospital, Zhejiang University School of Medicine, Hangzhou, 310009 China
Cardiovascular Key Laboratory of Zhejiang Province, Hangzhou, 310009 China
E-mail: hxy0507@zju.edu.cn

G. Liu, J. Xin, Prof. B. Wang
Cancer Institute (Key Laboratory of Cancer Prevention and Intervention, National Ministry of Education), The Second Affiliated Hospital, Zhejiang University School of Medicine, Hangzhou, 310009 China
E-mail: bwang@zju.edu.cn

G. Liu, J. Xin, Prof. Y. Feng, Prof. B. Wang
Institute of Translational Medicine, Zhejiang University, Hangzhou, 310029, China

Dr H. Zhu, Prof. W. Chen
Zhejiang University School of Medicine, Hangzhou, 310058 China

Prof. J. Zhang

Department of Biomedical Engineering, University of Alabama at Birmingham, AL, 35294 USA

Dr L. Zhang
College of Life Science, Zhejiang Chinese Medical University, Hangzhou, 310053 China.

Experimental methods

PAAM-mTG synthesis and characterization. The polysialic acid (PSA)-based anchor molecule (PAAM) was synthesized by Nanjing Goyoo Biotech (Nanjing, China). PSA (3 g) was dissolved in 100 mL anhydrous dimethylformamide (DMF) and 1 mL triethylamine under nitrogen, and the mixture was slowly warmed to 56 °C until the PSA was substantially dissolved; then, the solution was returned to room temperature, and 2-succinimido-1,1,3,3-tetra-methyluronium tetrafluoroborate (TSTU, 337 mg, 1.125 mmol) was added. The mixture was reacted at room temperature overnight; then, oleoylethylamine (396 mg, 1.125 mmol) was added, the reaction was continued for another 4 hours, and excess solvent was removed through reduced pressure distillation. Diethyl ether was added, and the resulting white powder was washed twice sequentially with dichloroethane and diethyl ether.

PAAM-mTG was synthesized by combining 0.2 mM PAAM with 0.2 mM mTG (Cool Seoul Bio) at room temperature in phosphate-buffered saline (PBS). Two hours later, the reaction mixture was transferred to an ultrafiltration centrifuge tube (Millipore, MW 50 kDa) and centrifuged at 4000 ×g and 4 °C for 30 min; then, the retention solution was washed twice and restored to a0.2 mM concentration in PBS, filtered with a syringe-driven filter unit (0.45 μm,

Millipore), and stored at 4 °C. The procedure was confirmed through^{1}H nuclear magnetic resonance (NMR) with a 600-MHz superconducting NMR spectrometer (Bruker, Avance-600).

Hydrogel synthesis and characterization. Gelatin (type A, 300 g bloom, Sigma-Aldrich) was dissolved in PBS (8%, w/v) at 60 °C; then, the solution was sterilized by filtration with a syringe-driven filter unit (0.45 μm, Millipore). For assessments in the absence of cells, the gelatin was crosslinked by combining 500 μL gelatin solution with 0.2 mM mTG in a 24-well plate for 30 minutes; then, the hydrogel was lyophilized and characterized by scanning electron microscopy (SEM, SU-8010, HITACHI) and solid-state NMR with a Bruker-Avance 400 III HD spectrometer (Bruker). NMR was conducted at a 9.7 T field strength and 100.61 MHz resonance frequency for ^{13}C with cross-polarization (CP), magnetic-angle spinning (MAS, 3.2-mm probe, 10-kHz spinning speed), and a high-power ^{1}H decoupling (2.4 ms^{1}H p/2 pulse, 2 ms CP pulse, 5 s recycle delay). The methylene signal of adamantane at 38.5 ppm was used as a reference for the ^{13}C chemical shift.

Hydrogel labeling for fluorescence. Fluorescein isothiocyanate (FITC) (150 μg) was dissolved in dimethyl sulfoxide (DMSO, 10 mg/mL) and added to 10 mL gelatin solution (5%

w/v in $NaHCO_3$ buffer, pH = 9.0); then, the mixture was stirred at 150 rpm and 37 °C for 6~8 h and dialyzed against deionized water for 2 days. The water was replenished every 4-6 h; then, the sample was lyophilized. All procedures were conducted in the dark, and the lyophilized product was dissolved in PBS (8%, w/v), filtered, and stored at 4 °C.

Sulfo-cyanine 7 (300 mg) was dissolved in DMSO (300 mg/mL) and added to 30 mL gelatin solution (5% w/v in Na_2CO_3 buffer, pH = 8.4); then, the mixture was stirred at 150 rpm and 20 °C for 5 h and dialyzed against PBS overnight. Then the mixture was dialyzed in pure water for 4 h and lyophilized finally. All procedures were conducted in darkness, and the lyophilized product was dissolved in PBS (8%, w/v), filtered, and stored at -20 °C.

MSC isolation and culture conditions. The tibias of 2-month-old male Sprague-Dawley (SD) rats (Slac Laboratory Animal, Shanghai, China) were rinsed with Dulbecco's modified eagle medium (DMEM, Thermo) to collect the bone-marrow cells; then, the cell mixture was centrifuged, the supernatant was discarded, and the cells were resuspended and cultured in MSC culture medium consisting of low glucose DMEM (90% v/v), FBS (10% v/v), and penicillin-streptomycin (1% v/v). Two or three days later, the medium was changed, the non-MSC population (i.e., the non-adherent cells) was removed, and the adherent cells were

cultured until a purified population of MSCs was obtained. MSCs were characterized by flow cytometry using specific cell surface antigens[S1].

Cells were maintained under standard culture conditions in low glucose DMEM and 10% fetal bovine serum under 21% oxygen and 5% carbon dioxide. Hypoxia was induced by replacing the standard medium with glucose- and serum-free medium (except as indicated) and incubating the cells for 24 hours in a ProOx-C–chamber system (Biospherix) under 0.5% O_2, 5% CO_2, and N_2. For TNFα treatment, the standard medium was replaced with optimal medium (Gibco) for 24 hours, and then with 100 ng/mL TNF-α in optimal medium for another 24 hours.

Nanogel-coated MSC generation. MSCs were cultured on a cell culture dish and trypsinized; then, 3.0-6.0×10^5 cells were suspended in 200 μL PBS containing PAAM-mTG (50-100 μM), cultured for 10-15 min at 37 °C, and centrifuged (1000 rpm, 3 min). The supernatant was removed; then, the MSCs were soaked in gelatin solution (8%, w/v) for 30 min at 37 °C, centrifuged (4000 rpm, 4 min) to remove excess gelatin solution, and rinsed with PBS. Formation of the nanogel coating was confirmed by laser scanning confocal microscopy (Nikon A1 Ti) of MSCs that had been coated with the FITC-conjugated gelatin

solution, and the encapsulation rate was determined by staining the cells with Hoechst 33342 (Aladdin) and evaluating FITC fluorescence through flow-cytometry analysis performed with a Beckman FC500 MPL instrument (Beckman).

Scanning electron microscopy (SEM). Nanogel-coated cells were fixed with 2.5% (v/v) glutaraldehyde overnight. The fixed cells were washed with 0.1 M phosphate-buffered saline (PBS) (pH 7.4) three times (15 min each). Then, cell samples were fixed with 1% osmic acid solution for 1.5 h and washed with 0.1 M PBS (pH 7.4) three times (15 min each). Subsequently, a series of ethanol gradient solutions with concentrations (v/v) of 30%, 50%, 70%, 80%, 90%, and 95% was used to dehydrate the fixed cells (15 min each) sequentially. Finally, the resulted cell samples were further dehydrated twice in 100% ethanol (20 min each). After that, the samples were dried and observed with a scanning electron microscope (Phenom Pro, Phenom-World, The Netherlands) with an accelerating voltage of 10 kV.

Size analysis of nanogel-coated MSCs. The size of nanogel-coated MSCs is measured by Mastersizer 3000 laser particle size analyzer (Malvern Instruments Ltd., Malvern Worcestershire, UK). The conditions of determination were as follows: 2.0 – 6.0×10^5 in 250

ml PBS, the pump set at 1000 rpm, and laser obscuration was sat at 10%. The sample was injected when the shading degree is between 0.1-15%.

Distribution and thickness of gelatin on the MSC surface. The MSCs were resuspended with electro-transfected buffer. Then mCherry plasmid (expressed in the cytoplasm) was added into the buffer and then the mixture was transferred into micropulser. The indicated program was used to transfer the plasmid into cells and these MSCs were plated on a dish for nanogel coating with FITC-labeled gelatin. After 8 hours, the distribution of gelatin in the MSC surface was observed via SIM microscopy (Nikon, Japan), then the image was processed by Imaris (Batch) 9.3.1. And the thickness of the coated nanogel was calculated by the homemade code with Image J software.

The pore size of gelatin hydrogel. PAAM-mTG (10 μM) was added into 20 mL gelatin solution (8%), then, the mixture was stirred at 150 rpm and 37 °C for 10 min and centrifuged at 3000 g to remove air bubbles. Pour mixture into 6 cm cell culture dish at 37°C. After 20 min, the gelatin was crosslinked, then the crosslinked gelatin was quickly frozen in liquid nitrogen for 10 min and then sublimated at room temperature for 15 s to sublimate all water inside the crosslinked gelatin. The freeze-dried hydrogel samples were then cut, fixed on

aluminum stubs, and coated with gold for 10 seconds for interior morphology observation with Cryo-scanning electron microscope (FIB Company, USA). The pore size distribution was determined by randomly selecting 300 pores from the FIB-SEM images.

Particle size distributions. The particle size distributions of isolated exosomes suspended in PBS was determined using ZetaView (Particle Metrix, Germany).

MSC mechanical properties. The mechanical properties of the cells were characterized through micropipette aspiration as described previously[S2]. Briefly, micropipettes with an inner diameter of 8-10 μm were created from borosilicate glass capillary tubes with a Flaming/Brown micropipette puller (P-1000, Sutter Instrument) and a microforge (Narishige), and the cells were trypsinized and suspended in warmed PBS. Suction pressure was applied through the capillary to the surface of the cell with a water negative pressure control system, and the equilibrium pressure (i.e., when the cell was neither aspirated nor pushed away from the micropipette) was recorded; then, the pressure was increased until the diameter of the cell was equal to the capillary diameter[S2a], and the pressure, the micropipette diameter, and the diameter of the portion of the cell that remained outside the pipette were recorded by the system software. Measurements were completed no more than 2 h after trypsinization, and 10

cells were measured for each experimental group or condition. Young's modulus and cortical tension were calculated according to the following equations (Tc: cortical tension; Pp: pipette pressure; Rp: pipette radius; Rc: cell radius; E: Young's modulus).

$$\mathrm{Tc} = \frac{\mathrm{Pp}}{2(\frac{1}{\mathrm{Rp}} - \frac{1}{\mathrm{Rc}})}, \qquad (\mathrm{Tc})\mathrm{equivalent} \approx 2.2\frac{\mathrm{ERp}}{1 - \left(\frac{\mathrm{Rp}}{\mathrm{Rc}}\right)} \approx 2.2\mathrm{ERp}$$

Binding force. The MSCs were suspended in DMEM buffer with 1% BSA, and red blood cells (RBCs) were fused to TNFα-biotin using a biotin protein labeling kit (Elabscience). Single MSCs and RBCs were gently aspirated with micropipettes and brought into contact with each other for 2 s; then, the cells were pulled apart with a pipette connected to a computer-controlled piezoelectric actuator. In the absence of adhesion, the RBC immediately separated from the MSC and returned to its original spherical shape, while in the presence of adhesion, the RBC remained bound to the MSC and was stretched into an elongated shape until separation. Eight to 12 pairs of cells were tested, and the cycle was repeated 50 times for each pair of cells.

Transmission electron microscopy (TEM). Cells (1×10^6) were suspended in double-distilled water, and the sample solution was dipped with a copper mesh supporting membrane. The membrane surface liquid was absorbed with filter paper; then, the membrane was dried

slightly, and 2% acetic acid uranyl uranium dye solution was added dropwise. The excess staining solution was removed with filter paper; then, the samples were dried and observed with a JEM 1200-EX transmission electron microscope (JEOL).

TEM was used to detect the mitochondrial length. In brief, 1×10^6cellswerefixed with 2.5% glutaraldehyde for 12 h. After washing three times with phosphate-buffered saline, the cells were post-fixed with 1% OsO_4 for 1–2 h. Next, the specimens were dehydrated by an ethanol gradient, followed by acetone for overnight infiltration. Furthermore, the specimens were embedded in Spurr resin and sectioned in Leica EM UC7 Manufacturer (Leica, Wetzlar, Germany). The sections were stained with uranyl acetate and alkaline lead citrate, and the images were obtained by Hitachi Model H-7650 TEMat6,800 × and 30,000 × magnification.

Cell apoptosis. Cells were cultured in 24-well plates (1×10^5 cells/well) under normal or hypoxic conditions for 24 hours, and then apoptotic cells were identified via deoxynucleotidyltransferase-mediated dUTP nick-end–labeling (TUNEL) with an In-Situ Cell Death Detection Kit (TMR red; Roche Applied Science, Indianapolis).

Tube formation. Tube formation was evaluated as described previously[S3]. Briefly, cells were plated in 24-well or 96-well plates (1×10^5or 2×10^4cells/well) that had been pre-coated

with growth factor-reduced Matrigel (BD) and incubated with the indicated medium for 4-6 h; then, tube length was quantified with Image-Pro Plus 6.0 software (Media Cybernetics). Five fields (100 × magnification) were counted in each well and3 wells in each group from three independent experiments were evaluated for each experimental group or condition.

Cell migration. Gel-coated and uncoated MSCs migration was performed in Transwell chemotaxis 24 well chamber (Corning). Cells (2×10^4 cells/well) in DMEM with 1% FBS were plated in the upper chamber, the lower chamber was filled with DMEM with 10% FBS. After 12 h, non-migrating cells were removed and washing with PBS. The migrated cells were fixed with 10% formaldehyde and stained with 0.1% crystal violet. Six fields were counted for each well (200 × magnification) and 3 wells in each group from three independent experiments, the migrated cells were counted by Image-pro plus 6.0 (Media Cybernetics).

Distribution of gelatin in MSC division. The FITC-labeled gelatin coated MSCs cultured incomplete medium with 10% FBS. Then, during MSCs was proliferation, the nanogel-coated MSC was observed every 10 min via laser scanning confocal microscopy (Leica, Wetzlar, Germany) to show the distribution of gelatin after cell division.

Proliferation. Cells were cultured in standard medium on 96-well plates at a density of 2×10^4 cells/well; then, the medium was replaced with 0.1 mL of DMEM containing 10% FBS, and the cells were placed in a humidified incubator maintained at 5% CO_2/95% room air and 37 °C. Cell proliferation was measured 24, 48, and 72 h later with a Universal Microplate Spectrophotometer (MD-SpectraMax M5) and a CCK-8 Kit as directed by the manufacturer's instructions.

MSC differentiation. Cells were seeded in a 6-well plate (1×10^5 cells/well) and differentiated into osteocytes, chondrocytes, and adipocytes via the protocols as described previously[S4].

Cellular ATP production. Cellular ATP content was measured by using an ATP determination kit (Beyotime Biotechnology) as directed by the manufacturer’s instructions.

Cellular oxygen consumption. Cellular oxygen consumption was measured with an Oxygraph-2k instrument (O2k; OROBOROS Instruments, Innsbruck, Austria)[S5]. Approximately 5×10^5cells were suspended in 2 mL of respiration media (0.5 mM EGTA, 3 mM $MgCl_2\cdot6H_2O$, 60 mM potassium lactobionate, 20 mM taurine, 10 mM KH_2PO_4, 20 mM HEPES, 110 mM sucrose, and 1 g/L fatty acid-free bovine serum albumin (BSA); pH 7.1)

and added to the chamber. The basal oxygen consumption rate was measured in the absence of substrates, and the maximum rate was measured while titrating 4 μM carbonyl cyanide 4-(trifluoromethoxy)-phenylhydrazone (Sigma).

Cytokine array. Cells (1×10^6) were cultured for 48 h with 10 mL of phenol red-free DMEM plus 10% FBS, and centrifuged for 3 min at 2500 rpm to remove cell debris; then, the proteins in the supernatants were quantified with the Rat Cytokine Array (RayBio, GSR-CAA-67), which can detect 67 proteins, via recommended protocols (Wayen Biotechnologies, Shanghai, China). Briefly, antibodies were printed onto slides to capture the corresponding cytokines, incubated with a mixture of biotinylated secondary antibodies, and labeled with Cy3-conjugated streptavidin; then, Cy3 fluorescence was detected with a GenePix 4000B Microarray Scanner (Axon), and the signal was digitized with GenePix Pro 6.0 software.

Western blot. Cells were rinsed with cold PBS, lysed in 2.5×sodium dodecyl sulfate (SDS) gel loading buffer (30 mM Tris-HCl, pH 6.8, 1% SDS, 0.05% bromphenol blue, 12.5% glycerol, and 2.5% mercaptoethanol) and boiled for 30 min; then, the proteins in the lysate were separated on 12% SDS polyacrylamide gels, electro-transferred to polyvinylidenedifluoride (PVDF) membranes (Millipore, Boston, MA), and stained with the

following primary antibodies: Bcl-2 (1:1000, Abcam), bak (1:1000, Servicebio), bax (1:1000, Abcam), TNFR1(1:1000, Proteintech), OPA1 (1:1000, CST), MFN1 (1:1000, Abcam), MFN2 (1:1000, CST), DRP1 (1:1000, CST), PCG1α (1:1000, Abcam), P-IκB (1:1000, CST), IκB (1:1000, CST), P-p50NFκB (1:1000, Abcam), p50NFκB (1:1000, Abcam), P-p65NFκB (1:1000,Abcam) and p65NFκB(1:1000, Abcam), VEAD (1:1000, CST), BNP (1:1000, Abcam), connexin43 (1:500, Abcam) and actin (1:3000, Kangcheng). Primary antibodies were stained with horseradish peroxidase-conjugated secondary antibodies and visualized with a chemiluminescence ECL Western-blotting system (Millipore).

Luciferase transfection. Cells (1×10^6) were infected with luciferase-encoding recombinant lentiviruses (Genechem Company) in 10 μg/mL polybrene (Millipore) for 12 h; then, the growth medium was replaced, and luciferase activity was detected by using an X Pack CMV-XP-Luciferase-EF1-Puro Expression Lentivector Kit (System bioscier) 48 h later.

SiRNA transfection. The TNFR-1, OPA1 and MFN2 siRNA was constructed by Ruibo Company, and the cells were transfected in 6-well plates (1×10^5 cells/well) for 48 h with a transfection kit (Ruibo). Transfected cells were cultured at 37 ºC in a 5% CO_2-humidified incubator.

Generation of conditioned medium. Cells were seeded in 6-well plates (1×10^5 cells/well) and cultured for 24 h; then, the medium was replaced with 2 mL of DMEM plus 10% FBS, and the cells were cultured for an additional 48 h. The conditioned medium was collected and centrifuged for 3 min at 2500 rpm to remove cell debris before use in subsequent experiments.

Isolation of neonatal rat cardiomyocytes. Hearts were extracted from 1- to 3-day-old neonatal rats (purchased from Zhejiang Chinese Medical University) and transferred into PBS. The tip of heart was cut into 1-mm^3 pieces, digested with 0.25% trypsin (Genom), and centrifuged for 10 min at 100 rpm; then, the supernatant was collected, and digestion/centrifugation was repeated until the tissue was fully digested. The collected supernatants were added to DMEM with 10% FBS, centrifuged for 10 min at 1500 rpm, and incubated for 1.5 h at 37 °C; then, the adherent cells (mostly fibroblasts) were discarded and the cardiomyocytes in the supernatant were collected.

MI model and treatment. Experiments involving live animals were performed in accordance with the Guide for the Care and Use of Laboratory Animals published by the US National Institutes of Health (NIH Publication No. 85-23, revised 1996) and were approved by the Institutional Animal Care and Use Committee of Zhejiang University. Male Sprague-Dawley

(SD) rats (250 g) were purchased from Zhejiang Chinese Medical University. The animals were fed a standard laboratory diet with free access to food and water and housed under a controlled temperature (22 ± 1°C) and humidity (65-70%) with a 12:12 h light : dark cycle. For MI induction, the animals were anesthetized via intraperitoneal injection of pentobarbital sodium (50 mg/kg) and ventilated via tracheal intubation and a rodent ventilator; then, the left anterior descending coronary artery was ligated with an 8-0 nylon suture. Treatments (1×10^6 MSCs or nanogel-coated MSCs in 100 µL PBS, or PBS alone) were administered 30 minutes later to five sites at the border of the infarction.

Echocardiography. Rats (number of animals in each group ≥ 8) were anaesthetized (3% sevoflurane mixed with 97% O_2) in an induction chamber, and transthoracic echocardiography was performed with a Vevo 2100Imaging System (Visual Sonics Inc). Left ventricular ejection fraction (EF) and fractional shortening (FS) were calculated from guided M-mode recordings as described previously[S6].

Bioluminescence. Animals (number of animals in each group ≥ 4) were anesthetized with pentobarbital sodium (i.p., 50 mg/kg) and intraperitoneally injected with D-luciferin (150

mg/kg in PBS, R&D); then, bioluminescence images were acquired over a 5-min period with an In vivo Imaging System (PerkinElmer).

Histological staining. Hearts were dehydrated in 30% sucrose solution, embedded in Tissue-Tek OCT compound, snap frozen in liquid nitrogen, and cut into 5-μm sections; then, the Masson's trichrome staining was performed to evaluate infract area by Masson's trichrome staining kit (Solaribio, Beijing, china). The images were measured using the Image-Pro Plus (Media Cybernetics). The infarct area was measured as the ratio (%) of the injured area divided by the whole left ventricular area. 4 animals were detected in each group and assessment of infarct size was performed in 5 sections per heart.

Immunohistochemical staining. Fixed cells or sections were washed with PBS, permeabilized with 0.2% Triton X (Sigma–Aldrich), and blocked with 5% bovine serum albumin. Apoptosis was evaluated with a TUNEL kit (Roche Applied Science). For immunofluorescence analyses, cells were incubated with primary antibodies against TMRM (Sigma), GFP (Abcam), CD31 (Chemicon), α-smooth muscle actin (α-SMA, Epitomics), phalloidin (Abcam) and PKH26 (Abcam), and with the corresponding secondary antibodies, and nuclei were counterstained with DAPI (Vector Laboratories, Burlingame, CA).

Real-time reverse-transcription polymerase chain reaction. Genomic DNA was obtained from cells and tissues with the TAKARA MiNiBEST Universal Genomic DNA Extraction Kit (TAKARA Biotechnology), and purified DNA was amplified with SYBR Premix Ex Taq (TAKARA Biotechnology). The amplification protocol was performed as described previously (S3). The engraftment rate was calculated according to the formula: engraftment rate = (number of GFP-positive cells per gram of heart tissue) × (mass of heart tissue) ÷ (number of cells administered) × 100%.

RNA sequencing. Total RNA was isolated from three replicates per experimental group or condition with a RNeasy Mini Kit (Qiagen) and sent to GENOME (Beijing, China) for preparation, sequencing, and mapping. The data were evaluated via hierarchical clustering analysis, pathway analysis, and cluster analysis.

Flow cytometry. ROS production was detected using an oxidation sensitive fluorescent probe 20, 70-dichlorodihydrofluorescein diacetate (DCFH-DA, Beyotime). Cells were incubated with 5 mM DCFH-DA for 30 min at 37 °C, then harvested and followed by flow cytometry with a BD FACS Count II Flow Cytometer (BD Biosciences, San Jose, CA, USA).

ELISA assay. ELISA kits were used to quantify IL-4 (mlbio), HGF (mlbio), PDGFAA (mlbio), LIX (mlbio), GM-CSF (mlbio), ICAM (mlbio), Neuropilin-2 (mlbio), B7-1 (mlbio). Conditioned medium was collected from MSC and Gel-MSC and analyzed according to the manufacturer's instruction.

Statistical analysis. Data are presented as the mean ± standard deviation (SD). Statistical significance was determined using one tailed t-tests for comparisons between two groups and one-way analysis of variance (ANOVA) and Tukey correction for comparisons among more than two groups. Analyses were performed with GraphPad Prism software (version 5.0), and $p < 0.05$ was considered statistically significant.

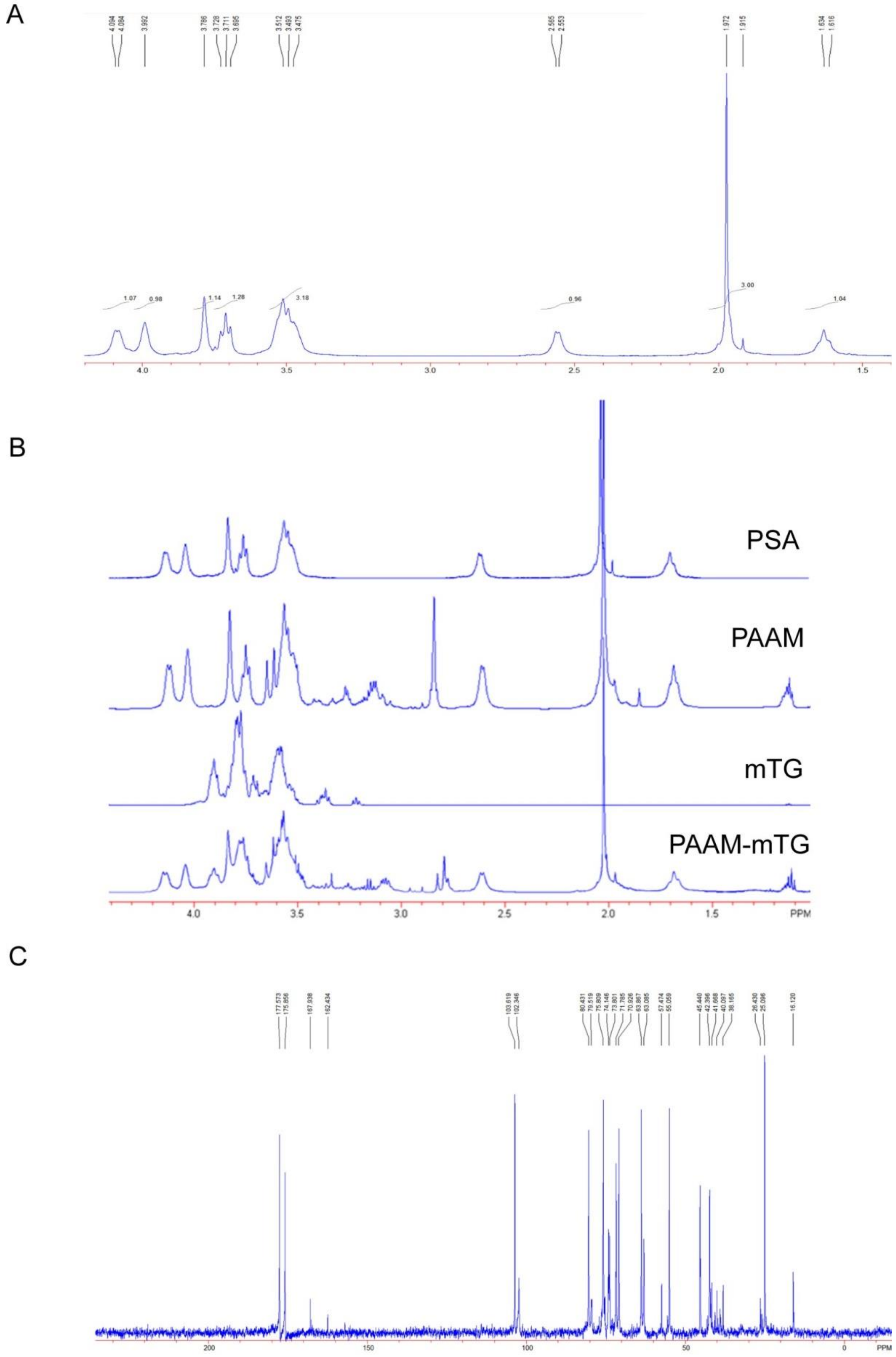

Figure S1. PAAM-mTG synthesis were confirmed via NMR. A)The^{1}H-NMR spectrum of PSA (D_2O, 600 M, 4.5-1.0 ppm). B) The structures of PSA, PAAM, mTG, PAAM-mTG(from

top to bottom) were evaluated via proton NMR. For the PAAM spectrum, the ethylenediamine peaks are at 3.61 and 3.65 ppm. The succinimide peak is at 2.8 ppm, and the alkane protons, which insert into the cell membrane are at 1.1-1.2 ppm. The succinimide peaks of PAAM decrease in the PAAM-mTG spectrum, and mTG peaks also are present in the PAAM-mTG spectrum. C) The ^{13}C-NMR spectrum of PAAM-mTG (D_2O, 600 M).

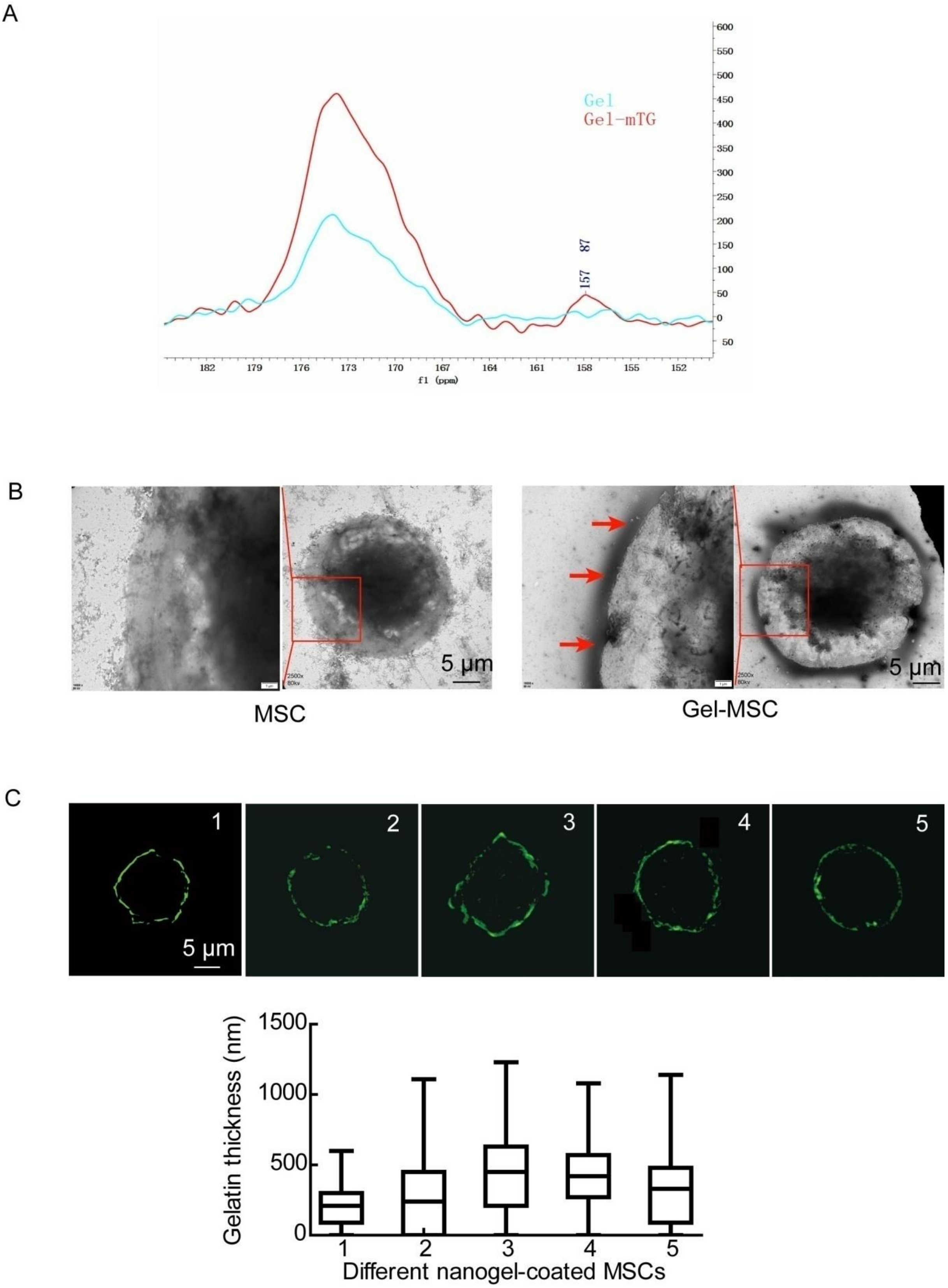
A
Gel
Gel-mTG
157.87
600
550
500
450
400
350
300
250
200
150
100
50
0
50
182
179
176
173
170
167
164
161
158
155
152
f1 (ppm)
B
5 μm
5 μm
MSC
Gel-MSC
C
1
2
3
4
5
5 μm
1500
1000
500
0
Gelatin thickness (nm)
1
2
3
4
5
Different nanogel-coated MSCs

Figure S2. Nanogel characterization. A) Nanogel polymerization were confirmed via solid-state NMR. The structure of the nanogel molecules before (blue) and after PAAM-mTG–induced polymerization (red) was evaluated via solid-state NMR. The labeled peak refers to the carbon which is in the amide bond newly formed via the mTG crosslinking reaction. B) The thickness of the nanogel coating was evaluated via transmission electron microscopy (TEM) (left: bar = 1 μm, right: bar = 5 μm). C)The thickness of the nanogel coating was evaluated via super-resolution structured illumination microscopy (Nikon SIM) (green: FITC-labeled nanogel; bar = 5 μm) and the thickness of the nanogel coating MSCs was measured every 5 degrees (right panel, n = 3), bar = 5 μm.

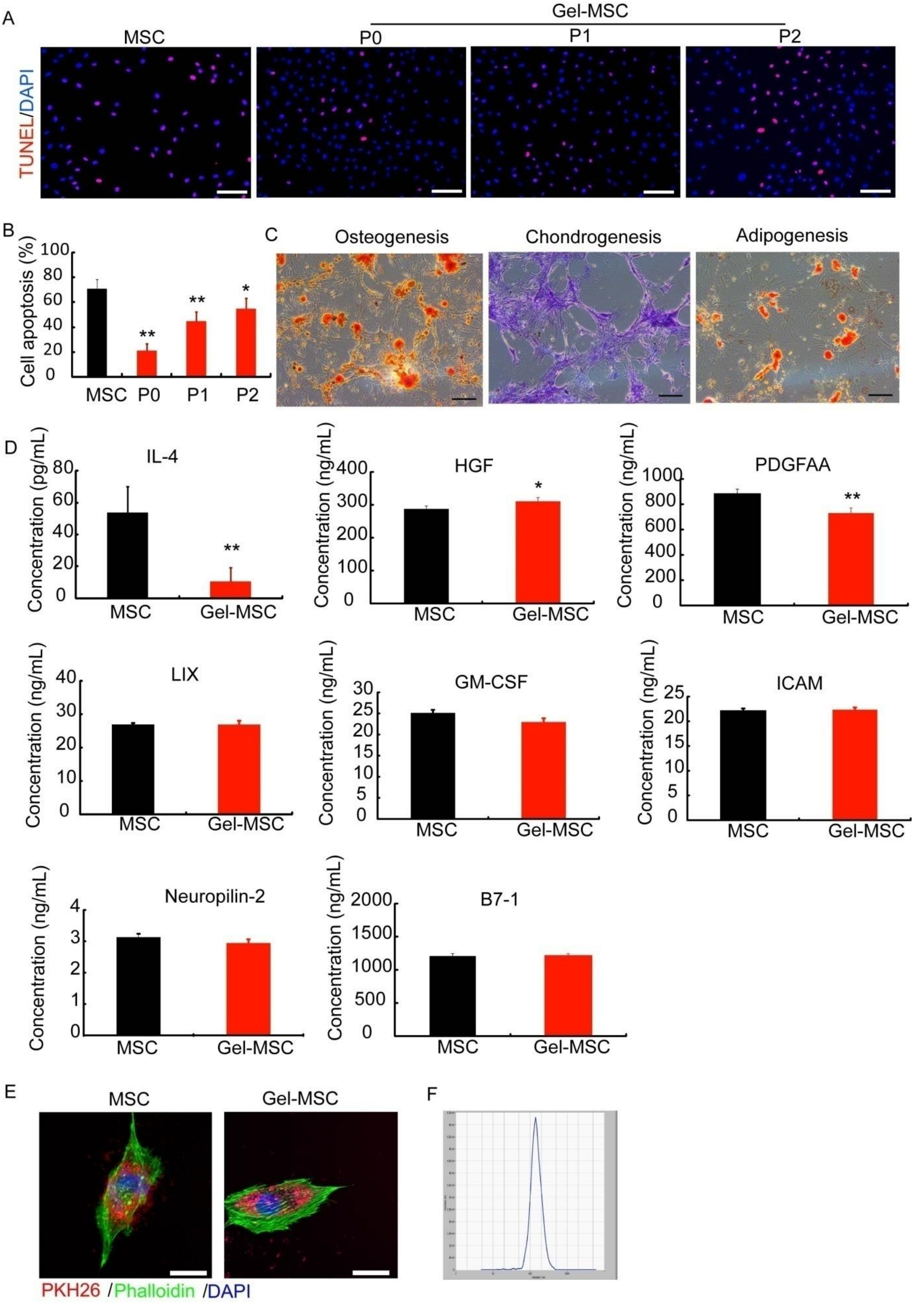

Figure S3. Single cell coating with surface-anchored nanogel does not impede MSC

protection against apoptosis and differentiation and the up- or down-regulated cytokines were validated. A) Cell apoptosis of the Gel-MSC (MSCs after nanogel coating) without cell passage (P0), with cell passage of once (P1), cell passage of twice (P2), cultured in serum- and sugar-free medium under hypoxic conditions, was detected by TUNEL (bar =100 μm). B) Apoptosis was quantified as the proportion of positively stained cells, n = 3 for three independent experimental repeats. C)Nanogel-coated MSCs were differentiated into osteocytes, chondrocytes, and adipocytes via established protocols; then, osteogenesis was evaluated with an alkaline phosphatase color development kit, chondrogenesis was evaluated via toluidine blue staining, and adipogenesis was evaluated by staining fat vacuoles with Oil Red O (bar = 100 μm). D)The up- or down-regulated cytokines were detected by ELISA. n = 3 for three independent experimental repeats, ** $p < 0.01$, * $p < 0.05$. E) Exosomes was taken by MSCs and nanogel-coated MSCs (Red: PKH26-labeled exosome, green: Phalloidin-labeled actin, blue: DAPI-stained nuclei; bar = 25 μm). F) The diameter of exosome was detected by ZetaView (Particle Metrix, Germany).

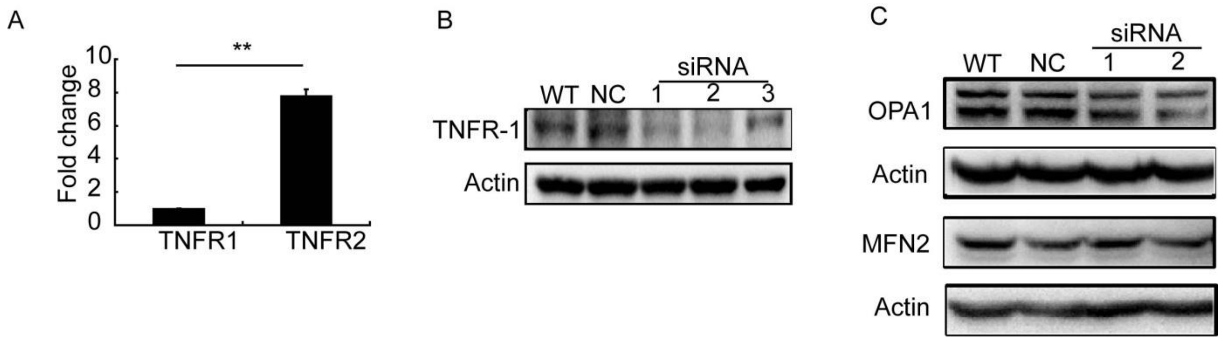

Figure S4. Gene expression and protein expression after transfecting with siRNA. A) Gene expression of TNFR1 and TNFR2 by qPCR, n = 3 for three independent experimental repeats, ** $P < 0.01$. B) TNFR1 protein levels were evaluated via Western blot in wild-type MSCs (WT), in MSCs transfected with nontargeting control siRNA (NC) and in MSCs transfected with TNFR1 siRNA (siRNA 1, 2, 3); actin levels were evaluated to confirm equal loading. OPA1 and MFN2 were required for protective effects of the cell surface nanogel coating by disrupting the binding of TNFα to TNFR in response to TNFα stimulation. C) OPA1 and MFN2 protein levels were evaluated via Western blot in wild-type MSCs (WT), in MSCs transfected with nontargeting control siRNA (NC) and in MSCs transfected with OPA1 (siRNA 1, 2) and MFN2 siRNA (siRNA 1, 2); actin levels were evaluated to confirm equal loading.

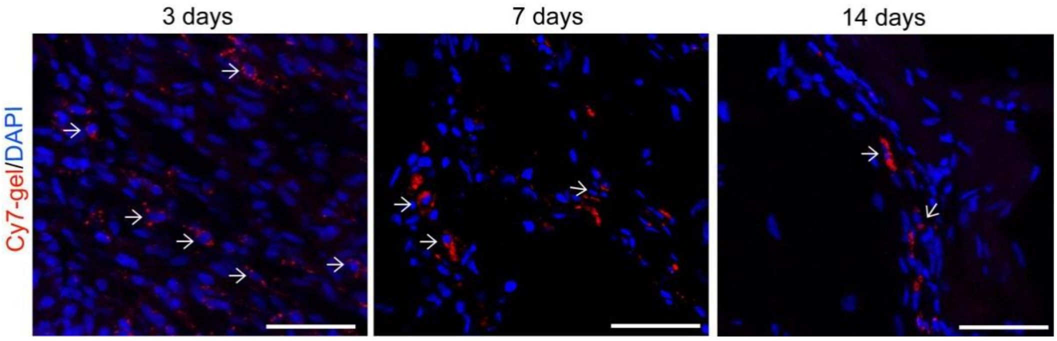

Figure S5. Sections of infarct heart after the transplantation of Gel-MSCs encapsulated with Cy7-labeled nanogel (red) were collected on day 3, 7 and 14 to identify nanogel coated MSCs. Bar=50 µm.

Supplementary References

[S1] H. Chen, X. Liu, W. Zhu, H. Chen, X. Hu, Z. Jiang, Y. Xu, L. Wang, Y. Zhou, P. Chen, N. Zhang, D. Hu, L. Zhang, Y. Wang, Q. Xu, R. Wu, H. Yu, J. Wang, *Front Aging Neurosci* **2014**, 6, 103.

[S2] a) H. Yu, C. Y. Tay, S. L. Wen, S. C. W. Tan, K. Liao, L. P. Tan, *Biochem Biophys Res Commun* **2010**, 393, 0; b) R. M. Hochmuth, *J Biomech* **2000**, 33, 15; c) W. Chen, J. Lou, E. A. Evans, C. Zhu, *J Cell Biol* **2012**, 199, 497.

[S3] H. Xu, P. Czerwinski, M. Hortmann, H. Y. Sohn, U. Forstermann, H. Li, *Cardiovasc Res* **2008**, 78, 349.

[S4] X. Hu, Y. Xu, Z. Zhong, Y. Wu, J. Zhao, Y. Wang, H. Cheng, M. Kong, F. Zhang, Q. Chen, J. Sun, Q. Li, J. Jin, Q. Li, L. Chen, C. Wang, H. Zhan, Y. Fan, Q. Yang, L. Yu, R. Wu, J. Liang, J. Zhu, Y. Wang, Y. Jin, Y. Lin, F. Yang, L. Jia, W. Zhu, J. Chen, H. Yu, J. Zhang, J. Wang, *Circ Res* **2016**, 118, 970.

[S5] B. Corradetti, F. Taraballi, J. O. Martinez, S. Minardi, N. Basu, G. Bauza, M. Evangelopoulos, S. Powell, C. Corbo, E. Tasciotti, *Sci Rep* **2017**, 7, 7991.

[S6] S. L. Puhl, A. Kazakov, A. Muller, P. Fries, D. R. Wagner, M. Bohm, C. Maack, Y. Devaux, *Br J Pharmacol* **2016**, 173, 88.
